# Follicular thyroid carcinoma with internal jugular vein tumour thrombus

**DOI:** 10.4314/gmj.v56i1.7

**Published:** 2022-03

**Authors:** Raphael N Mayeden, Klenam Dzefi-Tettey, Benard O Botwe

**Affiliations:** 1 Department of Radiology, School of Medicine, University of Health and Allied Sciences, PMB 31, Ho, Volta Region, Ghana; 2 Department of Radiology, Korle Bu Teaching Hospital. P. O. Box 77, Korle Bu, Accra, Ghana; 3 Department of Radiography, School of Biomedical & Allied Health Sciences, College of Health Science, University of Ghana, P.O Box KB 143. Korle Bu, Accra, Ghana

**Keywords:** Follicular carcinoma, thyroid gland, internal jugular vein, tumour thrombus, imaging

## Abstract

Tumour thrombus is the presence of tumour cells in great vessels. The reported incidence of tumour thrombus in thyroid carcinoma is about 0.2–3.8%. Being asymptomatic, detection of tumour thrombosis clinically is difficult. We present the report of internal jugular vein (IJV) tumour thrombosis in a known follicular thyroid carcinoma patient, detected with multimodality imaging. Grayscale ultrasound scan of the neck showed a well-defined, bi-lobed (2.4 x 1.5) cm, intraluminal solid lesion with homogeneous echotexture within the distal left IJV close to its confluence with the ipsilateral subclavian vein. The lesion showed significant internal vascularity on colour Doppler assessment. The sonographic findings confirmed further imaging with computed tomography (CT) and radioisotope scans. We conclude that patients with thyroid cancer should be evaluated for tumour thrombosis both clinically and with imaging, particularly with ultrasound and CT/MRI or nuclear medicine, as it has prognostic implications.

**Funding:**

None declared

## Introduction

Tumour thrombus is the presence of tumour cells in great vessels.^1^ Thyroid carcinoma with major cervical vascular tumour thrombosis is a very rare condition.^2^ The reported incidence of tumour thrombus in thyroid carcinoma is about 0.2 – 3.8%.^3^,^4^ The first reported cases of thyroid cancer tumour thrombus was by Kaufmann and Graham in 1879.^5^ Most often, tumour thrombus by thyroid carcinoma is asymptomatic,^6^ and may not be detected by clinical examination, not being palpable.^1^ Imaging plays a vital role in patient assessment and diagnosis.^1^,^6^

## Case Report

We report the case of a 68-year-old woman with a left internal jugular vein (IJV) tumour thrombus diagnosed by ultrasound and computerised tomography (CT) scan. The patient had had a total thyroidectomy at a peripheral hospital on account of multinodular goiter. Histopathology of the excised specimen showed minimally angio-invasive oncocytic follicular carcinoma of the thyroid gland Pt2 (calcitonin and CD 56 negative, TTF1, HMB1 positive on immunocytochemistry). An initial post-operative ultrasound scan done at the peripheral hospital suggested residual thyroid tissue in the left lobe of the thyroid gland. The patient was subsequently referred to the National Center for Radiotherapy and Nuclear Medicine [NCRNM], Korle Bu, Ghana, six weeks after the initial surgery for further management on account of the initial post-operative imaging findings and histopathologic results.

Physical examination by the Ear, Nose and Throat (ENT) team at the national referral centre was equivocal for any palpable anterior neck mass in the thyroid bed or the neck. Baseline thyroid function test (TFT) showed markedly elevated thyroglobulin levels – 23200mcg/L (upper limit 55mcg/L) and anti-thyroglobulin antibody level of 2.47mcg/L. A repeat ultrasound scan of the neck done as part of the work up for a possible completion thyroidectomy showed a well-defined, bi-lobed (2.4 x 1.5) cm, intraluminal solid lesion with homogeneous echotexture within the distal left IJV close to its confluence with the ipsilateral subclavian vein.

**Figure 1:**
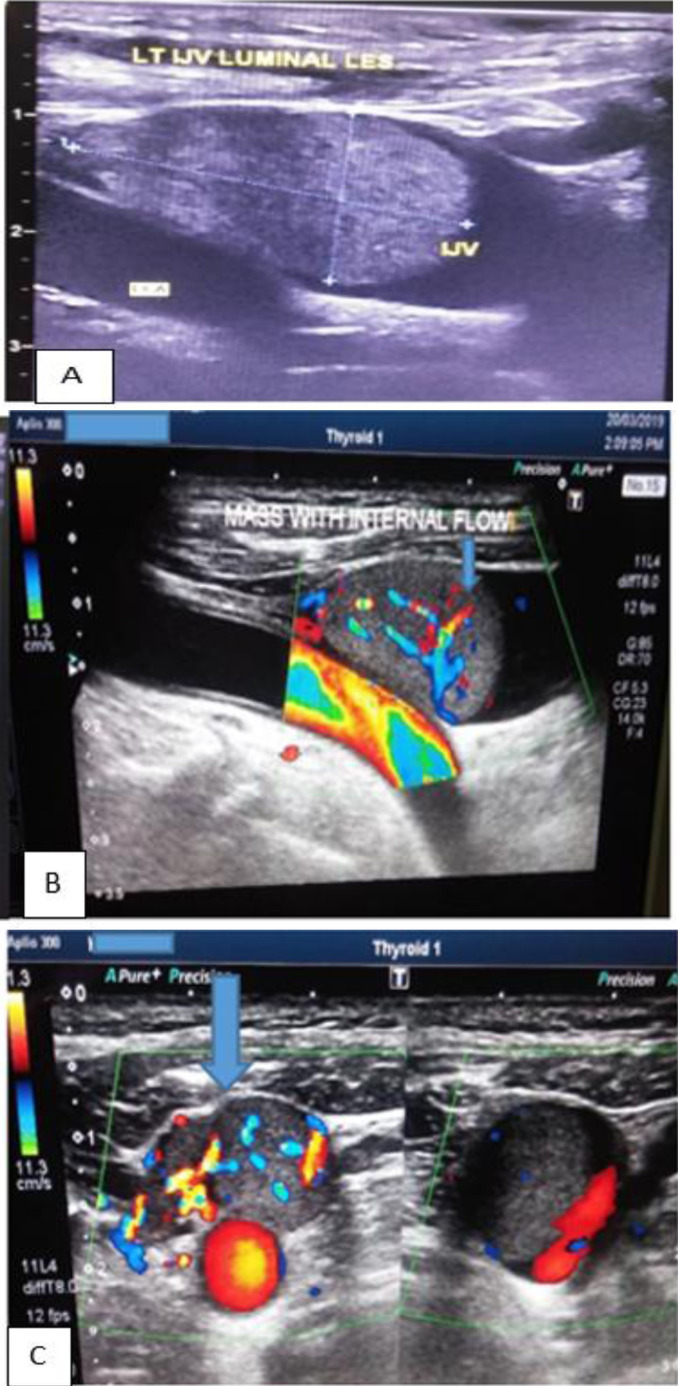
Grayscale (A) and colour Doppler ultrasound images (B and C) of the neck showing homogeneous left IJV intraluminal lesion (A) and significant internal vascularity (B & C).

The lesion showed significant internal vascularity on colour Doppler assessment (Figure 1). There was a minimal focal expansion of the left IJV but with intact walls. The thyroid bed was otherwise empty, with no residual extravascular thyroid tissue. No cervical lymphadenopathy was seen at the ultrasound. A conclusion of malignant left IJV tumour thrombus was made.

**Figure 2:**
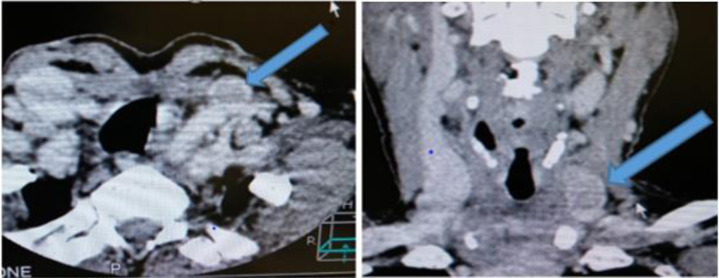
Contrast-enhanced CT scan showing a left IJV intraluminal lesion with a surrounding rim of contrast medium ‘the ring sign’ (arrows).

A subsequent CT scan of the head and neck showed a fairly defined filling defect with a surrounding rim of contrast medium (the ring sign) (Figure 2) in the distal left internal jugular vein close to the root of the neck. No other thyroid tissue/mass was seen at the CT scan. Follow-up TFT showed a marginal reduction in the thyroglobulin levels to 21,500mcg/L.

**Figure 3:**
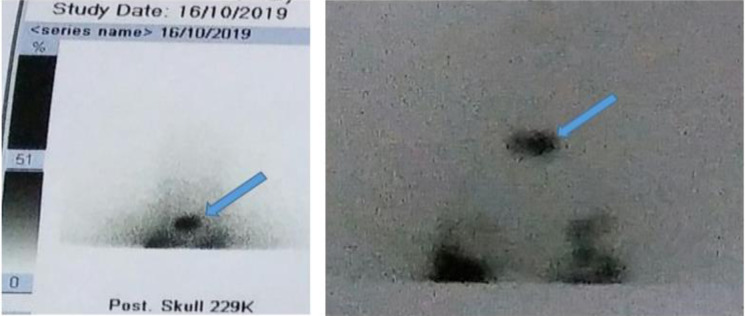
Iodine-131 radionuclide study showing uptake in supraclavicular region (Arrows).

The patient was optimised for radioactive iodine (RAI) therapy because of persistently high thyroglobulin levels with no palpable residual thyroid tissue. She received ablation with 100mCi of iodine-131 in the third month following the thyroidectomy. On post-therapy day 7, she underwent a whole-body scan, which revealed an area of activity in the supraclavicular region (Figure 3) – consistent with the root of neck IJV tumour thrombus and other areas of activity in the thoracic cavity and pelvis. The patient has shown a favourable response to therapy, evidenced by a serial reduction in serum thyroglobulin levels. She is currently being monitored on an outpatient basis. Consent for the case to be published (including images, case history and data) was obtained from the patient.

## Discussion

Even though carcinoma of the thyroid has the potential for microscopic vascular invasion, internal jugular vein (IJV) invasion by the tumour is a rare occurrence.^2^ The suggested mechanisms of spread are direct tumour thrombus, embolisation and infiltration with encasement.^7^ IJV invasion by thyroid carcinoma is a marker of the tumour's aggressiveness,^6^ and is generally a poor prognostic feature of any tumour.^8^ It is considered a risk factor for distant metastases (as in this case) or recurrence.^3^

Tumour thrombus is the presence of tumour cells in great vessels.^1^ Apart from thyroid carcinomas, renal cell carcinoma, renal transitional cell carcinoma, uterine sarcoma, Wilms' tumour, testicular tumour, adrenal cortical carcinoma, lymphoma, pancreatic cancer, osteosarcoma and Ewing's sarcoma are all known to cause vascular invasion.^9^–^11^

The reported incidence of tumour thrombus in thyroid carcinoma is about 0.2 – 3.8%.^3^,^4^ Papillary, follicular, Hurthle cell, insular and anaplastic thyroid cancers are subtypes of thyroid cancers with the potential for vascular invasion.^12^,^13^,^14^

Of all the subtypes of thyroid cancers, follicular thyroid cancers are notable for vascular tumour thrombus,^7^,^14^,^15^ as in this patient. Unlike papillary carcinoma, which spreads via the lymphatic route, follicular thyroid cancer spreads haematogeneously through the internal thyroid vein^14^,^16^, commonly, into the ipsilateral IJV, progressing either cephalad into the cranial vessels or caudad into the mediastinal vessels or the cardiac chambers.^8^

Most often, tumour thrombus by thyroid carcinoma is asymptomatic^6^ and may not be picked up by clinical examination, not being palpable.^1^ This patient had no clinical symptoms such as neck pain and swelling or a palpable mass on clinical examination. Often, serum thyroglobulin levels are markedly elevated^17^,^18^,^19^ as in this case and can be used as a marker for surveillance in the post-treatment period if available.^14^

Radiological imaging has a vital role in the management of vascular tumour thrombus. The condition may be accidentally diagnosed on imaging or as part of the routine peri-operative radiological evaluation.^1^,^6^ Various imaging modalities exist for evaluating patients with suspected tumour thrombus. In the index case, ultrasound scan of the neck as part of the work-up for a possible completion thyroidectomy was the initial imaging study, even though the diagnosis was missed on the initial study.

The ultrasound scan was the only modality readily available at the peripheral facility of the various imaging modalities. Ultrasound scan of the neck is generally routinely used,^1^,^4^,^8^,^15^ being widely available and non-invasive.^15^ It is useful in characterising the tumours and identifying the location of extrathyroid masses^4^,^15^ and for diagnosing small vascular tumour thrombi.^8^ As seen in this case, the tumour thrombus shows internal vascularity on colour Doppler, differentiating it from bland thrombus. The usefulness of high-frequency Doppler ultrasound as a non-invasive method for detecting tumour extensions into the great veins have been demonstrated in a case series.^20^ The limitation of ultrasound is that it is operator-dependent, and the diagnosis may be missed if a skilled person does not perform it.

Computed tomography (CT) scan is another imaging modality for diagnosing thyroid tumour thrombus.^1^,^6^,^18^ Contrast-enhanced CT scan of the head and neck shows the tumour thrombus as a filling defect in the vessel, sometimes with a rim of contrast around the filling defect, the so-called ring sign.^14^ The CT scan on our case demonstrated this sign (Figure 2.0). The presence of this sign is an indication of non-adherence to, or non-invasion of the tumour into the vascular wall and, therefore, the feasibility of transcervical thrombectomy.^14^,^20^ According to some authors, CT scan is the best imaging modality for detecting tumour thrombus in thyroid cancer.^1^,^7^,^19^ It is an important factor in the clinical outcome.^19^ Unfortunately, the scarcity of this imaging modality, added to the cost of the procedure, limits its use in most peripheral facilities in Ghana.

RAI can be used to differentiate a tumour thrombus from a bland venous thrombus, in that the former is generally iodine-avid,^18^ as was seen in this case. Other modalities that have been used in literature to evaluate tumour thrombus are magnetic resonance imaging (MRI) and fluorine-18 fluorodeoxyglucose positron-emission tomography (18-FDG/PET).^1^,^21^The lack of evidence-based guidelines makes management challenging.^15^ Multimodality regimen comprising surgery, external beam radiotherapy (EBRT) and RAI therapy has been recommended to treat aggressive thyroid cancer with great vein invasion.^17^ Some authors suggest that complete surgical resection has a better outcome, without which there is drastic deterioration in patients' quality of life.^15^,^21^ The decision to manage the patient with RAI was based on the persistently high thyroglolin levels. High serum thyroglobulin levels are an indication for RAI therapy. Nakashima et al. 17 reported a significant decline from a very high serum thyroglobulin level to a normal range in a patient following RAI therapy. Patient response has also been reported in EBRT.^17^,^21^ In this patient, RAI therapy was both diagnostic and therapeutic. The patient showed a favourable response to the RAI.

## Conclusion

Patients with thyroid cancer should be evaluated for tumour thrombosis prior to and after surgery. Post-operative imaging procedures are important when a malignant lesion is suspected.
